# The effect of oral sildenafil therapy on health-related quality of life in adults with pulmonary arterial hypertension related to uncorrected secundum atrial septal defect: a quasi experimental study

**DOI:** 10.1186/s12955-020-01498-7

**Published:** 2020-08-14

**Authors:** Fera Hidayati, Putrika P. R. Gharini, Anggoro Budi Hartopo, Dyah Wulan Anggrahini, Lucia Kris Dinarti

**Affiliations:** Cardiology and Vascular Department, Faculty of Medicine, Public Health and Nursing, Universitas Gadjah Mada - Dr. Sardjito Hospital, Yogyakarta, Indonesia

**Keywords:** Health-related quality of life, Pulmonary arterial hypertension, Atrial septal defect, Sildenafil

## Abstract

**Background:**

Assessment of health-related quality of life (HRQoL) are often measured as an important patient-reported outcome (PRO) in clinical studies. Pulmonary arterial hypertension (PAH) is a common complication of atrial septal defect (ASD). This study aimed to compare the HRQoL of PAH related uncorrected secundum ASD at pre and post therapy with oral sildenafil therapy.

**Methods:**

We conducted quasi experimental study at Sardjito General Hospital Yogyakarta since April 2016 to August 2017. Adults with PAH related uncorrected secundum ASD, listed on Congenital Heart Disease and Pulmonary Hypertension (COHARD-PH) registry, and met the inclusion and exclusion criteria were recruited as subject. Interview was done at pre and 12 weeks post oral sildenafil therapy 3 × 20 mg using the EQ-5D-3L questionnaire. Statistical analysis was done using Wilcoxon test and paired T-test to determine the differences of EQ-5D utility and EQ-VAS score at pre and post therapy.

**Results:**

A total of 18 adult patients with PAH related to uncorrected secundum ASD were enrolled in this study (83.33% female; mean age 38.72 ± 10.81 years old). The most frequent reported problems pre therapy were pain/discomfort (83%) and anxiety/depression (78%). Wilcoxon test showed the median of EQ-5D utility score were increased after sildenafil therapy (before = 0.604, after = 0.664; Z = − 2703; p:0.007), respectively. Meanwhile, the paired T-test results showed an increase of EQ-VAS mean difference 6.67 ± 8.75 (p:0.005; 95% CI 2.32–11.02) after sildenafil therapy.

**Conclusion:**

The administration of oral sildenafil therapy 3 × 20 mg during 12 weeks in adult patients with PAH related uncorrected secundum ASD gives better HRQoL.

## Backgrounds

Pulmonary Artery Hypertension (PAH) is a common complication of congenital heart disease which mostly happen on patients with left to right shunt, such as atrial septal defect (ASD). Uncorrected left to right shunt may increase pulmonary pressure and lead to vascular remodeling and dysfunction. Thus, it is responsible for progressive increase of pulmonary vascular resistance and right heart pressure [1]. Based on disease registry of PAH on ASD patients in Sardjito General Hospital Yogyakarta, out of 123 adult ASD patients, 74% among them had PAH [2]. Post (2013) mentioned that PAH was found in 9–35% secundum ASD, including those who have or have not corrected [3].

PAH symptoms such as activity-induced dyspnea, dizziness, cough, chest pain, palpitation, and peripheral edema may impact the physical mobility and emotional status that could worsen the patients’ health related quality of life (HRQoL) [4]. HRQoL is a parameter of personal satisfaction in living affected by health status, such as physical capacity, cognitive ability, working relations, emotional and spirituality. It is subjective, multidimensional, and temporary [5]. To date, only few data showed a potential output from patient-reported outcome (PRO) in showing the prognosis of PAH. PRO is a patient’s health parameter measured by themselves which includes HRQoL, that is a functional effect of disease and therapy consequences by patient’s judgement [6].

Meta-analysis of 4 researches on safety and efficacy of sildenafil therapy for > 12 weeks in patients with PAH concludes that sildenafil significantly decrease clinical deterioration events and increase 6 min’ walk test distance, WHO functional class, hemodynamic parameters and HRQoL compared to placebo [7]. HRQoL improvement has been reported in PAH patients with the specific therapy, but it does not show consistency in all of the researches [8–11]. Thus, we aim to investigate whether there are HRQoL differences before and after sildenafil therapy in adult patients with PAH related uncorrected secundum ASD.

There are many disease-specific questionnaires for PAH such as Cambridge Pulmonary Hypertension Outcome Review (CAMPHOR), emPHasis-10, Minnesota Living with Heart Failure Questionnaire (MLHFQ), Pulmonary Arterial Hypertension Symptom Scale (PAHSS), and Living with Pulmonary Hypertension (LPH) [12]. However, we use EQ-5D-3L questionnaire as a tool to assess HRQoL because its serve the aim of self-completion, only need a few minutes to complete, has a value set which can be used to evaluate quality-adjusted life-years (QALY), and we obtain freely access to this questionnaire from EuroQol [13]. The EQ-5D-3L questionnaire also has been proven to be valid and reliable in Indonesian population [14].

## Methods

### Study design

We conducted a quasi-experimental research in Sardjito General Hospital Yogyakarta from April 2016 to August 2017. The subject included in this research was adult patients (age ≥ 18 years) with PAH and uncorrected secundum ASD who had been registered on COHARD-PH registry and signed the informed consent. Secundum ASD was diagnosed by trans thoracic echocardiography and trans esophageal echocardiography, meanwhile PAH was diagnosed by right heart catheterization. Exclusion criteria were not completing 12-weeks follow up, other congenital heart defect, WHO NYHA functional class I, had received specific therapy for PAH, pregnancy, had received nitrates, or chronic pulmonary diseases.

Demography and clinical data; such as age, gender, WHO functional class, marital status, comorbid disease, and other therapy; were recorded in case report form. Subject filled HRQoL questionnaire before and 12 weeks after receiving oral sildenafil 3 × 20 mg. The therapy adherence was done by multiple phone call reminder and two-weeks-routine follow up to the hospital. Side effect evaluation and dose adjustment toward the clinical condition were done in every routine follow-up meeting.

Instrument to measure HRQoL was generic questionnaire EuroQol-5 Dimensions 3 Levels (EQ-5D-3L) which had been proven validity and reliability. This questionnaire was developed by the EuroQol Group which consist of 2 parts; EQ-5D descriptive system and visual analogue scale (EQ-VAS).

The descriptive system assesses patients’ quality of life in 5 dimensions which are mobility, self-care, usual activities, pain/discomfort, and anxiety/depression. Based on the response level in each EQ-5D-3L dimension, subjects will be categorized into no-problem group, moderate-problem group, and severe-problem group.

The response of each dimension in the EQ-5D descriptive system will be converted into single summary index based on valuation techniques that will create a value set for each level in the index. The values are adjusted differently in various countries and related to a health state [13]. The index ranged between 0 and 1 [15]. A value of 0 is equivalent to being dead while a value of 1 means the healthiest condition imaginable. The Malaysia value set was used in this research. The valuation technique used to estimate the Malaysia EQ-5D value set was Time Trade-off (TTO) and Visual Analogue Scale (VAS) [16]. To date, there are no value set of EQ-5D-3L available for Indonesia. Endarti (2016) stated that Malaysia value set is preferable to use in Indonesian population compared to another value set [17].

The EQ-VAS is a self-rated measurement which concerned to imaginable health state by subject with score range 0–100 [13].

The difference of EQ-5D utility score and EQ-VAS before and after therapy will be analyzed further.

#### Sample size

Using the formula to determine minimum sample for numerical analysis research and considering the 10% drop-out rate, we found that estimated sample needed was 19 subjects. The ‘standard deviation of the mean’ and ‘x_1_-x_2_’ value was adapted from the research conducted by Peppe-Zaba [8].

#### Echocardiography evaluation

Trans thoracic echocardiography was performed by experienced technician and verified by cardiologist consultants. Bubble test was performed when interatrial defect was not clear on echocardiography examination. Bubble test and trans esophageal echocardiography was conducted by cardiologist consultant.

#### Right cardiac catheterization

Right cardiac catheterization was performed by a cardiologist through standard operating procedure in Sardjito General Hospital Yogyakarta, using angiography machine Xper Cardio Physiomonitoring 5 hemodynamic monitors (Philips, USA). Saturation and pressure were measured on every location using oximeter (Avoximeter® 1000E, USA).

#### Questionnaire EQ-5D-3L data sampling

Questionnaire data sampling was performed by trained enumerator. Data sampling was performed through (1) Subject was given a thorough explanation about the questionnaire filling, (2) Subject filled the questionnaire, (3) Subject was allowed to ask questions, (4) Subject could be assisted on reading and filling questionnaire, if needed.

### Statistical analysis

Statistical analysis was performed using SPSS for Windows 22.0. Continuous variables are presented as mean ± standard deviation, while categorical variables are presented as percentages. Comparative analysis for paired categorical variables (before and after therapy) was performed for every EQ-5D dimension using McNemar and Wilcoxon test. Comparative analysis to compare the EQ-5D utility score and EQ-VAS before and after therapy was performed using Wilcoxon signed rank test and paired T-test, respectively. Consequent analysis was performed to find the correlation between factors that influence difference between utility score of EQ. 5D and EQ-VAS. The analysis was performed using Mann Whitney test due to abnormal data distribution. *p* value < 0,05 was considered statistically significant.

## Results

A total of 22 patients were included in the study subjects. During the study, 4 subjects were dropped out (one subject died in a local hospital about a week after starting sildenafil therapy, two subjects did not continue the study at the 4th week due to loss of follow up, and one subject only followed the study until the 8th week because of health insurance issues) and were not analyzed further. We could not determine the patient’s cause of death because she died in another hospital. But, based on the follow up by phone call that the patients felt shortness of breath, we assumed that the cause of death was related to the PAH complication rather than the sildenafil effect. We obtained 18 patients who could follow the study for 12 weeks. During the study, there were 2 subjects (11.11%) who reported dizziness after taking sildenafil 3 × 20 mg. Because of that, the sildenafil dose was reduced to 2 × 20 mg and the dizziness improved. This is considered a mild side effect which improved after dose reduction.

Subjects were predominantly women with 83.33% (15) subjects being women. Mean age of subjects was 38.72 ± 10.81 years old. All of the subjects were symptomatic, 72.22% (13) subjects were in WHO functional class II and 27.78% (5) were in WHO functional class III. The majority of subjects were married (83.33%), 3 of whom had no children and 2 were widowed. 11 (61.11%) subjects were housewives.

Other comorbid diseases were present in 2 subjects (11.11%) with one subject diagnosed with atrial fibrillation and one subject diagnosed with ischemic heart disease, whereas 16 subjects (88.89%) were not accompanied by other diseases. A total of 10 subjects (55.56%) did not receive supportive PAH therapy (furosemide, digoxin, spironolactone, warfarin) and 8 subjects (44.44%) treated with supportive PAH therapy. Table 1 showed basic characteristics of PAH subjects with uncorrected ASD II who received sildenafil 3 × 20 mg for 12 weeks.

**Table 1:** Baseline characteristic of subject

Variable		Value (***n*** = 18)
Age (years)	Mean ± SD	38.72 ± 10.81
Gender	Female, n (%)	15 (83.33)
	Male, n (%)	3 (16.67)
WHO Functional Class	II, n (%)	13 (72.22)
	III, n (%)	5 (27.78)
Marital Status	Not married, n (%)	3 (16.67)
	Married, with children, n (%)	10 (55.56)
	Married, no children, n (%)	3 (16.67)
	Widowed, n (%)	2 (11.11)
Occupation	Housewives, n (%)	11 (61.11)
	Civil servant, n (%)	2 (11.11)
	Private employee, n (%)	3 (16.67)
	College student, n (%)	1 (5.56)
	Unemployed, n (%)	1 (5.56)
Comorbid disease	None, n (%)	16 (88.89)
	Present, n (%)	2 (11.11)
Other therapy	No therapy, n (%)	10 (55.56)
	Furosemide, n (%)	8 (44.44)
	Digoxin, n (%)	7 (38.89)
	Spironolactone, n (%)	1 (5.56)
	Warfarin, n (%)	1 (5.56)
	Aspirin, n (%)	1 (5.56)

*SD* Standard Deviation

**Fig. 1:**
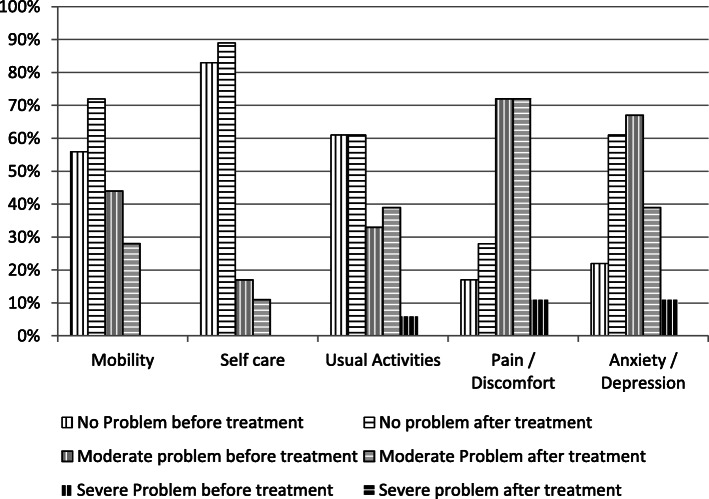
Comparison of health status based on five-dimensional EQ-5D in adult patients with PAH due to uncorrected secundum ASD at the time before and after sildenafil therapy 3 × 20 mg for 12 weeks

The subjects were further categorized into three groups; which were no-problem group, moderate-problem group, and severe-problem group at the time before and after sildenafil therapy based on the response level of EQ-5D-3L questionnaire. Figure 1 showed that the most reported problems were pain/discomfort (72% moderate and 11% severe problems), followed by anxiety/ depression (67% moderate and 11% severe problems), and usual activities limitations (33% moderate problems and 6% severe problems). After receiving 3 × 20 mg sildenafil therapy for 12 weeks, those severe problems were no longer reported by the subject.

**Fig. 2:**
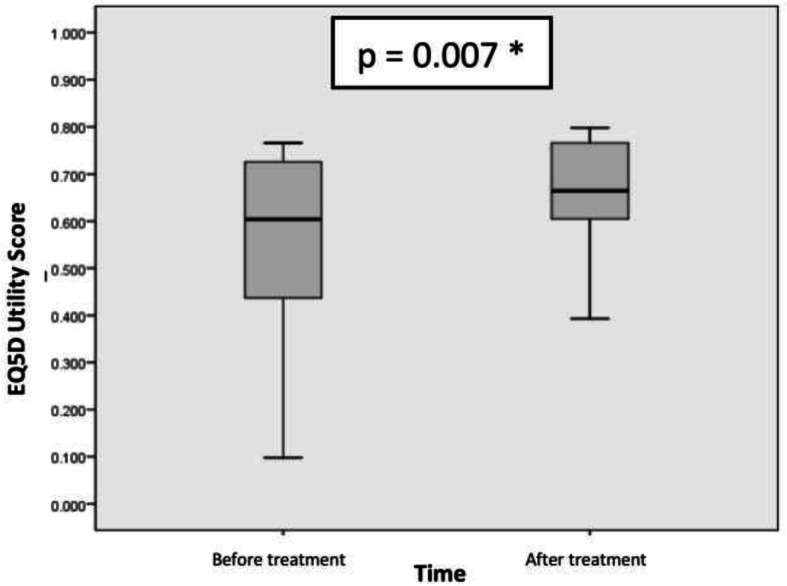
The median of EQ-5D utility score before and after 12 weeks of oral sildenafil therapy 3 × 20 mg in adult patients with PAH due to uncorrected secundum ASD proved to be statistically significant. * *p* < 0.05

**Fig. 3:**
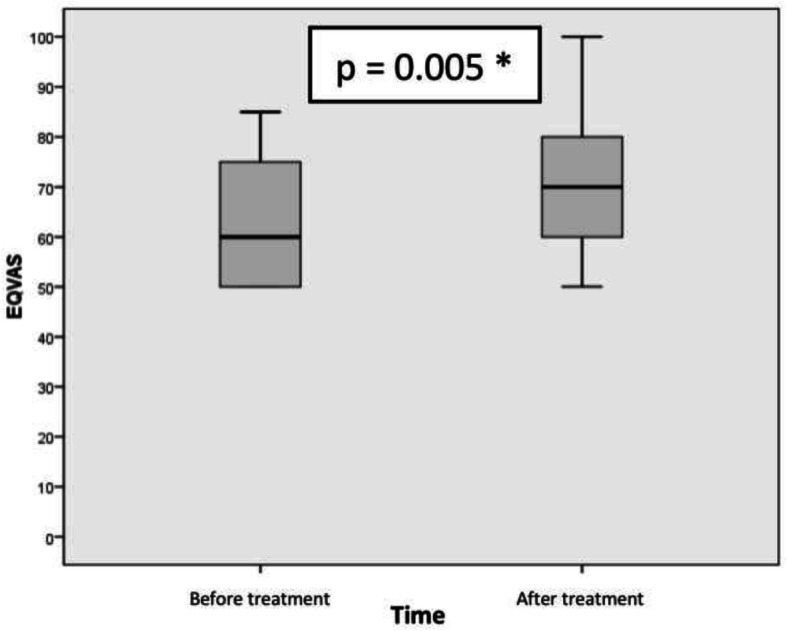
The mean of EQVAS score before and after 12 weeks of oral sildenafil therapy 3 × 20 mg in adult patients with PAH due to uncorrected secundum ASD proved to be statistically significant. * *p* < 0.05; 95% CI

Figures 2 and 3 showed the difference of EQ-5D utility set and EQ-VAS before and after sildenafil therapy. Comparative analysis before and after therapy was performed using Wilcoxon test for EQ-5D utility score and paired T test for EQ-VAS. The result was shown in Table 2. The median of EQ-5D utility score were increased after therapy (before = 0.604, after = 0.664; Z = − 2.703, p:0,007), while the mean of EQ-VAS score also were increased 6.67 ± 8.75 (95% CI, 2.32 to 11.02) *p*-value 0.005.

**Table 2:** EQ5D utility score and EQ-VAS score analysis

Time	EQ5D Utility Score				
	Median	Minimum	Maximum	Z-score	*p*
Before treatment	0.604	0.098	0.766	−2.703	0.007*
After treatment	0.664	0.393	0.798		
Time	EQ-VAS Score				
	Mean (SD)	Difference (SD)	Mean 95% CI		*p*
			Lower	Upper	
Before treatment	65 ± 13.06	6.67 ± 8.75	2.32	11.02	0.005*
After treatment	71.67 ± 15.53				

*SD* standard deviation, *EQ5D* EuroQoL-5 Dimensions, *EQVAS* EuroQoL-Visual Analogue Scale, *p < 0.05; 95% CI

Thus, there were statistically significant result in both median difference of EQ-5D utility score and mean difference of EQ-VAS before and 12 weeks after oral sildenafil therapy.

The subjects studied were the same subjects who performed HRQoL measurements twice, before and after therapy. Factors influencing changes in EQ-5D and EQ-VAS utility scores were analyzed by re-grouping subjects based on these factors, such as sex group (male and female), age (> 38 years and ≤ 38 years), education level (low and high), marital status (married and not married), employment status (employed and unemployed), other therapies (with and without other therapies), and comorbidities (with and without coexisting illness).

There was an increase of EQ-5D and EQ-VAS utility score after oral sildenafil therapy in all of the sub groups. The EQ-5D and EQ-VAS utility score differences were calculated in each sub-group and were analyzed using Mann Whitney test due to abnormal data distribution. *p* value < 0.05 is considered statistically significant. From Table 3, there was no statistically significant difference between all factors affecting HRQoL to the difference of EQ-5D and EQ-VAS utility scores.

**Table 3:** Factor affecting EQ-5D utility score and EQ-VAS score difference

Variable	Utility Score Difference	*P*	EQ-VAS Score Difference	*P*
Gender				
Male, *n* = 3	0.011 ± 0.018	0.083	11.67 ± 7.64	0.242
Female, *n* = 15	0.001 ± 0.109		16.67 ± 5.77	
Age				
< 38 y.o, *n* = 9	0.089 ± 0.114	0.656	4.44 ± 9.17	0.119
≥ 38 y.o, *n* = 9	0.122 ± 0.210		10 ± 7.50	
Education status				
Low, *n* = 2	0.254 ± 0.530	1.000	15 ± 7.10	0.166
High, *n* = 16	0.062 ± 0.042		6.25 ± 8.47	
Marital Status				
Married, *n* = 15	0.052 ± 0.034	0.339	13.33 ± 11.55	0.460
Not Married, *n* = 3	0.185 ± 0.160		5 ± 13.23	
Occupation				
Employed, *n* = 5	0.027 ± 0.045	0.091	9 ± 7.42	0.443
Unemployed, *n* = 13	0.494 ± 0.460		4 ± 8.9	
Other treatment				
With treatment, *n* = 8	0.192 ± 0.208	0.089	5 ± 9.64	0.249
No treatment, *n* = 10	0.051 ± 0.050		8.13 ± 8.43	
Comorbid disease				
With comorbid disease, *n* = 2	0.099 ± 0.140	0.944	2.5 ± 3.54	0.511
Without comorbid disease, *n* = 16	0.106 ± 0.171		10 ± 14.14	

Description: Mann Whitney test analysis results showed *p* value> 0.05 in all sub groups. This means that there is no statistically significant difference between the factors affecting HRQoL to the difference of EQ-5D utility score and EQ-VAS score, * *p* < 0.05

## Discussion

Based on the data, we found that the mean age of subjects diagnosed as PAH in uncorrected secundum ASD was 38.72 years. These results are in accordance with the study of Haque et al. (2015) which stated that PAH development in secundum ASD mostly occurred in the third decade [18]. Vogel et al. (1999) noted that the incidence of PAH in secundum ASD were increased in patients at the age of 18 to 40 year old [19]. The majority of the subjects of this study were female (83.33%) which similar to the previous study [19]. Euro Heart Survey registry also support this findings. They concluded that the incidence of PAH in female ASD was 76.6%, higher than male patients [20].

All subjects have many problems in various EQ-5D dimensions before starting the specific PAH therapy. In general, subjects reported their best performance on the self-care dimension and their worst on pain/discomfort dimension. Severe problems experienced by 11% of subjects on both pain/discomfort and anxiety/depression dimensions, while 6% of the subjects felt severe problems on performing usual activities. This result is in accordance with the study by Thompson et al. (2001) in Germany who found that the best performance of both primary and secondary PAH patients was experienced in the self-care dimension while the worst performance was reported on the dimension of usual activities. In this study, 20% of subjects experienced severe problems while performing usual activities and less than 10% experienced severe problems in other dimensions [21]. Mychaskiw et al. (2010) who examined the health status of PAH patients in the subjects of SUPER 1 clinical trial found that moderate to severe problems mostly occur in the usual activity dimension (77%), while the least occur in self-care dimension (24%) [22].

After 12 weeks of treatment, severe-problem response level was no longer experienced in the dimensions of pain/discomfort, anxiety/depression, and usual activities. The percentage of subjects who did not had any complain in all of the 5 EQ-5D dimensions were increased. The severity of each EQ-5D dimension problem were decreased. In line with these results, Pepke-Zaba et al. (2008) proved in his study that improvement was achieved in all dimensions of EQ-5D after 12 weeks of sildenafil therapy [8].

A clinical trial by Pepke-Zaba et al. (2008) found that the mean of EQ-5D utility score changed 0.10 ± 0.04 (*p* < 0.01) and the mean of EQ-VAS changed 8 ± 2 (*p* < 0.01) [8]. Our study showed similar changes that after sildenafil therapy, the median of EQ-5D utility score was increased after therapy (before = 0.604, after = 0.664; Z = − 2.703, p:0,007), while the mean of EQ-VAS score also were increased 6.67 ± 8.75 (95% CI, 2.32 to 11.02) *p*-value 0.005.

Determination of drug effects on HRQoL is an important component in evaluating the effect of drugs on clinical outcomes and health care. Several previous studies that evaluated HRQoL after sildenafil administration showed consistent results with this study. A study in patients with primary pulmonary hypertension (*n* = 22) conducted by Sastry et al. (2004) mentioned that there was an increase in dyspnea and fatigue components of heart failure questionnaire after receiving 12 weeks sildenafil therapy (dose: 3 × 25 mg, 3 × 50 mg and 3 × 100 mg) [23]. Another study by Wong et al. (2007), involving 19 HAP patients (idiopathic, connective tissue disease, and CHD, also evaluated HRQoL changes 3 months after sildenafil therapy (dose: 3 × 25 mg and 3 × 50 mg) using SF-36 questionnaire. There was an increase on physical, social, and general health score [24]. Tay et al. (2011) used CAMPHOR questionnaire to evaluate HRQoL in 12 patients with Eisenmenger syndrome who were given sildenafil 3 × 20 mg. The result showed an improvement in HRQoL after 3 months of therapy [25].

Although the drug administration significantly improves the EQ-5D based on statistics, the clinical benefit can be trivial for patients. Thus, the score difference must exceed the MCID (Minimal Clinical Important Difference) that represent the minimal amount of benefit that were recognized by the patient [26]. A review of 18 studies conducted by Coretti et al. (2014) stated that MCID for EQ-5D index ranged from 0.03 to 0.52 [26]. The EQ-5D median difference from this study is 0.06 which concluded that sildenafil therapy gave a clinically significant benefit for the patients. However, none of the 18 studies used in the review were conducted in patients with cardiac problem. To the best of our knowledge, there are no studies assessing MCID for EQ-5D index in cardiac problem patient population. Other than that, we believe that the result can be used as a treatment consideration for pulmonary arterial hypertension cases especially in developing countries.

Demographic factors such as age, sex, marital status, education level, and employment status have an influence on HRQoL. A systematic review by Gu et al. (2016) who assessed factors affecting HRQoL of PAH patients explained that HRQoL is influenced by demographic characteristics (such as living alone, decreased social support), mental health (such as anxiety, depression, stress), physical health (such as exercise and symptomatic capacity), and pharmacologic therapy [4]. In this study, the utility score mean of subjects aged < 38 years was lower than subjects aged ≥38 years, but the mean of EQ-VAS score was the opposite (*p* > 0.05). Study by Matura et al. (2014) that examined the difference in symptoms severity and HRQoL of young, middle, and older PAH patients concluded that the decrease in HRQoL component was experienced by all age groups, but the younger age group had slightly better physical function compared to other groups [27]. Symptom severity, therapy complexity, or psychological stressors made the patients hard to work. This led to job losses, disrupted economic conditions and social isolation [28].

Nilsson (2012) mentioned that in a group of unmarried / single patients had a low HRQoL [29]. Similarly, this study found that the mean of both utility scores and EQ-VAS of unmarried subjects were lower than those married subjects (*p* > 0.05). Living alone and minimal social support worsen HRQoL emotional dimension score. In contrast, working actively is associated with a better HRQoL physical dimension score [4]. The mean of both utility scores and EQ-VAS in unemployed subgroup in this study, were lower than those that worked (*p* > 0.05).

Delcroix and Howard (2015) published an article review on the burden of PAH disease and its impact on quality of life. It was explained that PAH patients who aged > 50 years old were reported to have more comorbid diseases such as ischemic heart disease, coronary artery disease, hypertension, atrial fibrillation, diabetes, and hypothyroidism than younger patients. The presence of comorbidities results in delayed diagnosis of PAH in older patients. The higher burden of comorbidities also contributes to the lower survival rates of older PAH patients in the UK and Ireland, which is about 3 times higher than the younger population (≤50 years) [30]. In line with this, in this study, the mean of EQ-5D utility score and EQ-VAS of subjects with comorbidities were lower than those without comorbidities (*p* > 0.05).

Supportive therapies (such as diuretics, digoxin, oral anticoagulants, or oxygen) is one of the PAH patient’s management strategies mentioned in the ESC guidelines on PAH (2015). Right heart failure leads to fluid retention, increased central venous pressure, hepatic congestion, ascites, and peripheral edema. Clinical experience shows the benefits of diuretics to reduce fluid retention symptoms, but no randomized trials related to diuretic use in PAH patients. Diuretic therapy is recommended in PAH patients with signs of right heart failure and fluid retention, with recommendation class Ic. Aldosterone antagonist therapy may be considered, along with plasma electrolyte levels and renal function monitoring, to prevent hypokalemia and pre-renal kidney disease [31].

High prevalence of vascular thrombotic lesions was found on postmortem examination of idiopathic PAH patients. In addition, the abnormalities of the coagulation and fibrinolysis cascades in the PAH patient population have also been reported. Oral anticoagulants are administered to PAH patients with consideration of increased risk of venous thromboembolism (heart failure and immobilization). The benefits of oral anticoagulant therapy are limited to idiopathic PAH, hereditary PAH, and anorexigen-related PAH (recommendation class IIb). This is largely derived from retrospective single center studies. In a contrary, the research result conducted from registry and randomized clinical trials are heterogenous and inconclusive [31]. Current evidence of anticoagulant drugs’ efficacy and safety in PAH patient populations is limited. The clinical guidelines do not recommend routine anticoagulant treatment in Eisenmenger syndrome patients and suggest to give anticoagulant treatment in atrial fibrillation and pulmonary artery thrombosis without major haemorrhage [32].

In this study 44,44% (8) subjects received other therapy, including PAH support therapy. A subject (5.56%) was treated with oral furosemide if necessary, six (33.34%) subjects treated with oral furosemide 1 × 20 mg and oral digoxin 1 × 0.125 mg, and a subject (5.56%) with accompanying atrial fibrillation received oral furosemide 1 × 40 mg, digoxin 1 × 0,125 mg, spironolactone 1 × 25 mg, and warfarin 1 × 2 mg. The mean of EQ-5D utility score and EQ-VAS in group of subjects receiving other therapies were lower than without comorbidities (*p* > 0.05). This is in line with cross-sectional studies by Zlupko et al (2008) that evaluate HRQoL PAH patients with various etiologies such as idiopathic, familial, systemic sclerosis, CHD, human immunodeficiency virus (HIV), liver disease, anorexigen, and obstructive pulmonary venous disease. A total of 93 subjects who were recruited received epoprostenol therapy (28%), bosentan (49%), calcium channel blockers (47%), sildenafil (3%), digoxin (33%), diuretics (57%) and warfarin (49%). The HRQoL evaluation was measured by a specific PAH disease questionnaire MLHF-PH. The study found a severe HRQoL disorder in all subjects. There were no HRQoL differences in patients treated with diuretics, digoxin, and oxygen [33].

## Conclusion

This study concluded that oral sildenafil therapy 20 mg three times per day for 12 weeks in PAH patients due to uncorrected secundum ASD significantly improve HRQoL.

## Data Availability

Data can be shared upon contact with the correspondence author.

## Abbreviations

ASD: Atrial Septal Defect
CAMPHOR: Cambridge Pulmonary Hypertension Outcome Review
CI: Confidence Interval
CHD: Congenital Heart Disease
DIG: Digitalis Investigation Group
EQ-5D-3L: EuroQoL-5 Dimensions-3 Levels
HIV: Human Immunodeficiency Virus
HRQoL: Health-Related Quality of Life
MLHFQ: Minnesota Living with Heart Failure Questionnaire
NYHA FC: New York Heart Association Functional Class
PAHSS: Pulmonary Arterial Hypertension Symptom Scale
PRO: Patient-Reported Outcome
VAS: Visual Analog Scale
WHO: World Health Organization
